# Classic Ewing Sarcoma of the Ankle With Ewing Sarcoma RNA-Binding Protein 1-Friend Leukemia Integration 1 (EWSR1/FLI1) Fusion: A Case Report and Review of Diagnostic Considerations

**DOI:** 10.7759/cureus.101021

**Published:** 2026-01-07

**Authors:** Yanina Nikolaus, Gabriel Urbelz

**Affiliations:** 1 Pathology, Marshall University Joan C. Edwards School of Medicine, Huntington, USA; 2 Internal Medicine, Marshall University Joan C. Edwards School of Medicine, Huntington, USA

**Keywords:** ankle mass, ewing sarcoma, ewsr1/fli1, molecular pathology, small round blue cell tumor

## Abstract

Ewing sarcoma (ES) is a malignant small round blue cell tumor most frequently arising in the long bones of adolescents and young adults, characterized by a translocation involving the Ewing sarcoma RNA-binding protein 1 (*EWSR1*) gene, most commonly the *EWSR1*/friend leukemia integration 1 (*FLI1*) fusion. We report a case of a 26-year-old man with left ankle pain found to have an extraosseous soft tissue mass surrounding the calcaneus. Histopathology revealed a highly cellular neoplasm of uniform small round blue cells arranged in sheets and nests, immunoreactive for cluster of differentiation 99 (CD99) with diffuse membranous staining and negative for AE1/AE3, desmin, CD45, and S100. Targeted next-generation sequencing confirmed an *EWSR1*/*FLI1* fusion, establishing the diagnosis of ES.

## Introduction

Ewing sarcoma (ES) is a highly aggressive malignant neoplasm composed of undifferentiated small round cells with characteristic rearrangements involving the Ewing sarcoma RNA-binding protein 1 (*EWSR1*) gene, most commonly the *EWSR1*/friend leukemia integration 1 (*FLI1*) (t(11;22)(q24;q12)) fusion. ES typically arises in the diaphysis of long bones, most commonly the femur, tibia, and humerus, or in the pelvis and predominantly affects children and young adults; however, it may also occur in extraosseous soft tissue sites.

Given the morphologic overlap with other small round blue cell tumors, including *CIC*-rearranged sarcoma, *BCOR*-rearranged sarcoma, lymphoma, and rhabdomyosarcoma, immunohistochemistry and molecular testing are essential for definitive diagnosis. Here, we report a molecularly confirmed case of classic ES arising in the extraskeletal soft tissues of the ankle in a young adult man, a highly uncommon anatomic site. This case underscores the importance of maintaining diagnostic consideration for Ewing sarcoma at rare extraskeletal locations and highlights the value of integrated histopathologic, immunohistochemical, and molecular evaluation in establishing the diagnosis in such unusual presentations.

## Case presentation

A 26-year-old man presented with progressive left ankle pain without associated systemic symptoms, including fever, weight loss, or night sweats. His history was notable for a remote left ankle twisting injury sustained while playing basketball at age 14, with complete clinical resolution at that time and no chronic symptoms thereafter.

**Figure 1:**
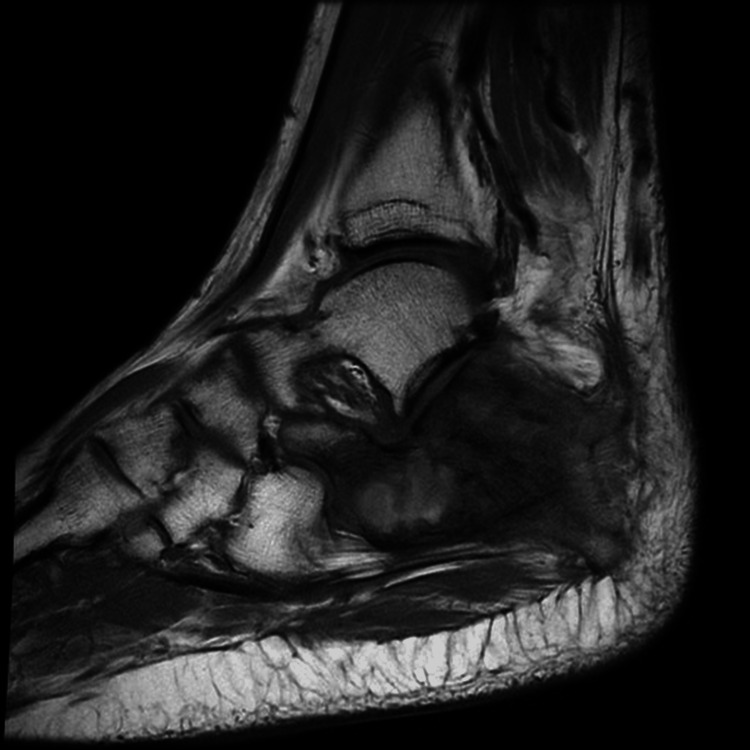
MRI of the left ankle (sagittal view) showing an extraosseous soft tissue mass surrounding the calcaneus.

MRI with contrast (09/20/2025) demonstrated an extraosseous soft tissue mass surrounding the calcaneus, measuring 4.6 × 2.9 cm laterally and 4.1 × 1.8 cm medially (Figure 1). Laboratory evaluation revealed leukocytosis and a markedly elevated erythrocyte sedimentation rate (ESR), findings that may be seen in ES despite its malignant nature (Table 1).

**Table 1:** Selected laboratory findings at presentation.

Laboratory Test	Result	Reference Range
White blood cell count	13.76 × 10⁹/L	4.0-11.0 × 10⁹/L
Erythrocyte sedimentation rate (ESR)	97 mm/hour	<20 mm/hour

**Figure 2:**
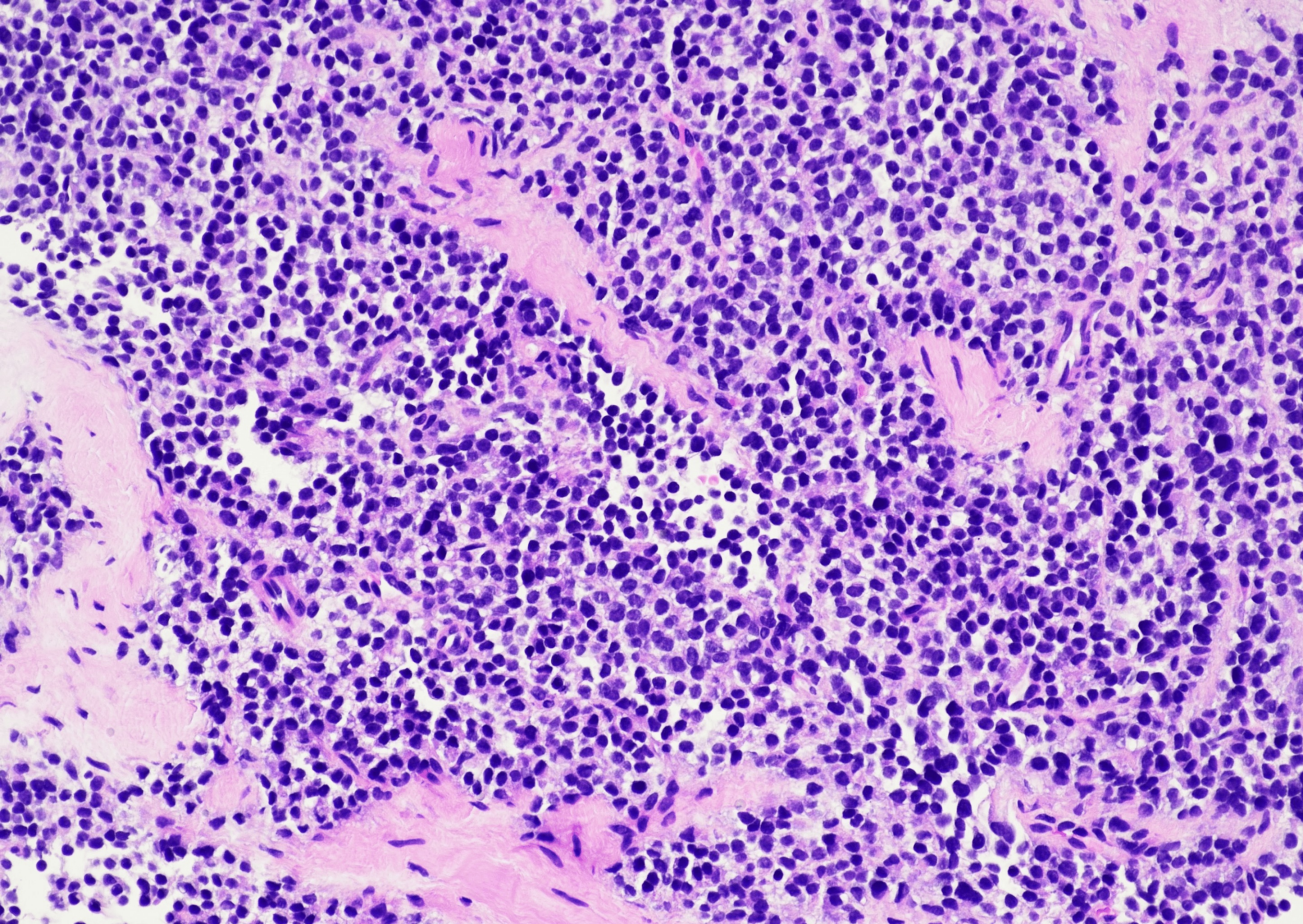
A highly cellular small round blue cell neoplasm with focal Homer-Wright rosette formation on H&E stain at 20×. H&E: 20×. Highly cellular neoplasm composed of uniform small round blue cells arranged in solid sheets within soft tissue. Focal Homer-Wright rosette formation is present (top right quadrant), characterized by tumor cells arranged around central fibrillary material.

**Figure 3:**
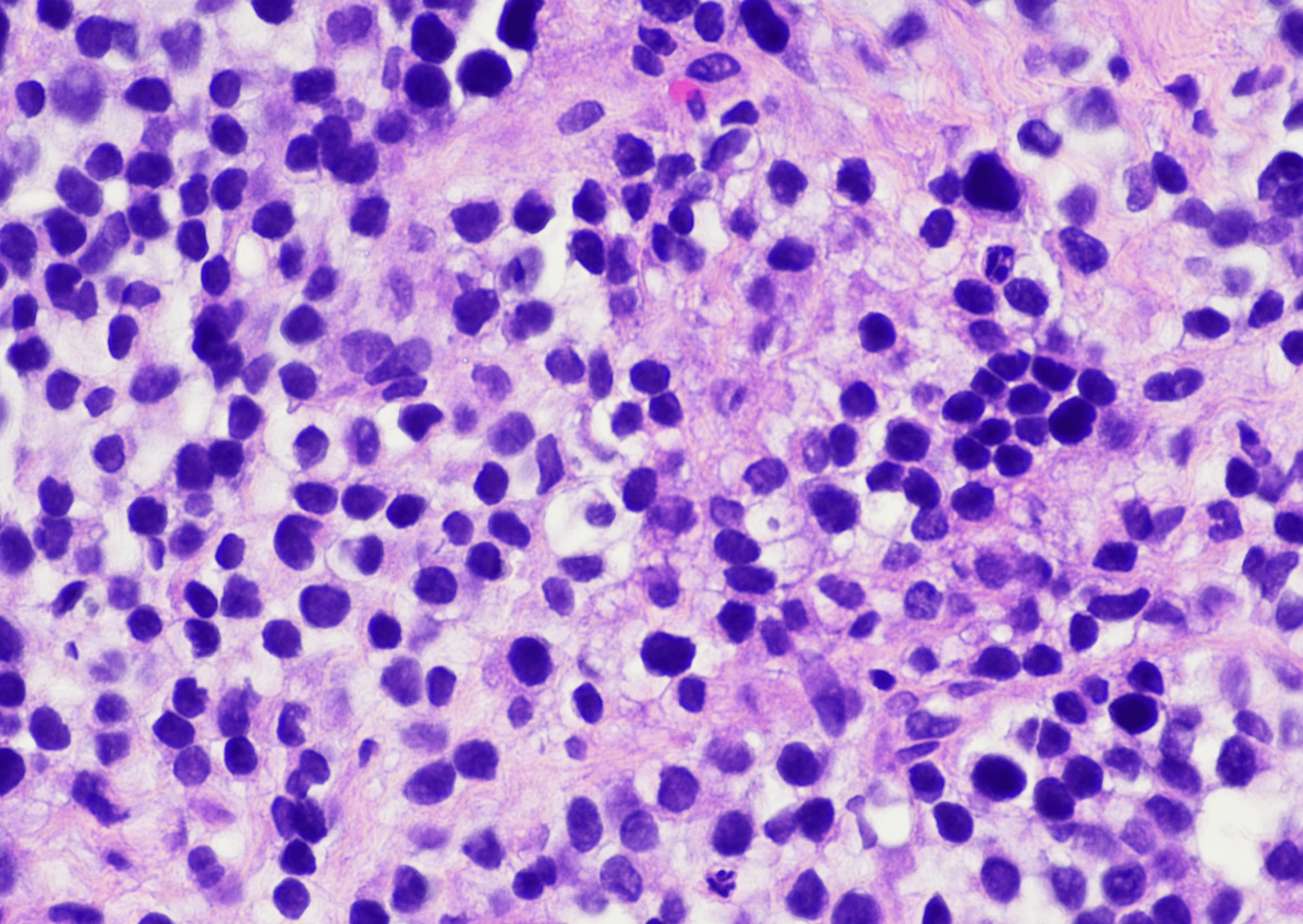
Higher magnification (×400 total magnification) showing uniform tumor cells with scant to moderate pale, vacuolated cytoplasm and finely dispersed chromatin. H&E stain: ×400 total magnification. Tumor cells show uniform round nuclei with finely dispersed chromatin and inconspicuous nucleoli. While the cytoplasm is classically scant in Ewing sarcoma, many cells in this field exhibit a moderate amount of pale to vacuolated cytoplasm, a feature consistent with intracytoplasmic glycogen accumulation.

**Figure 4:**
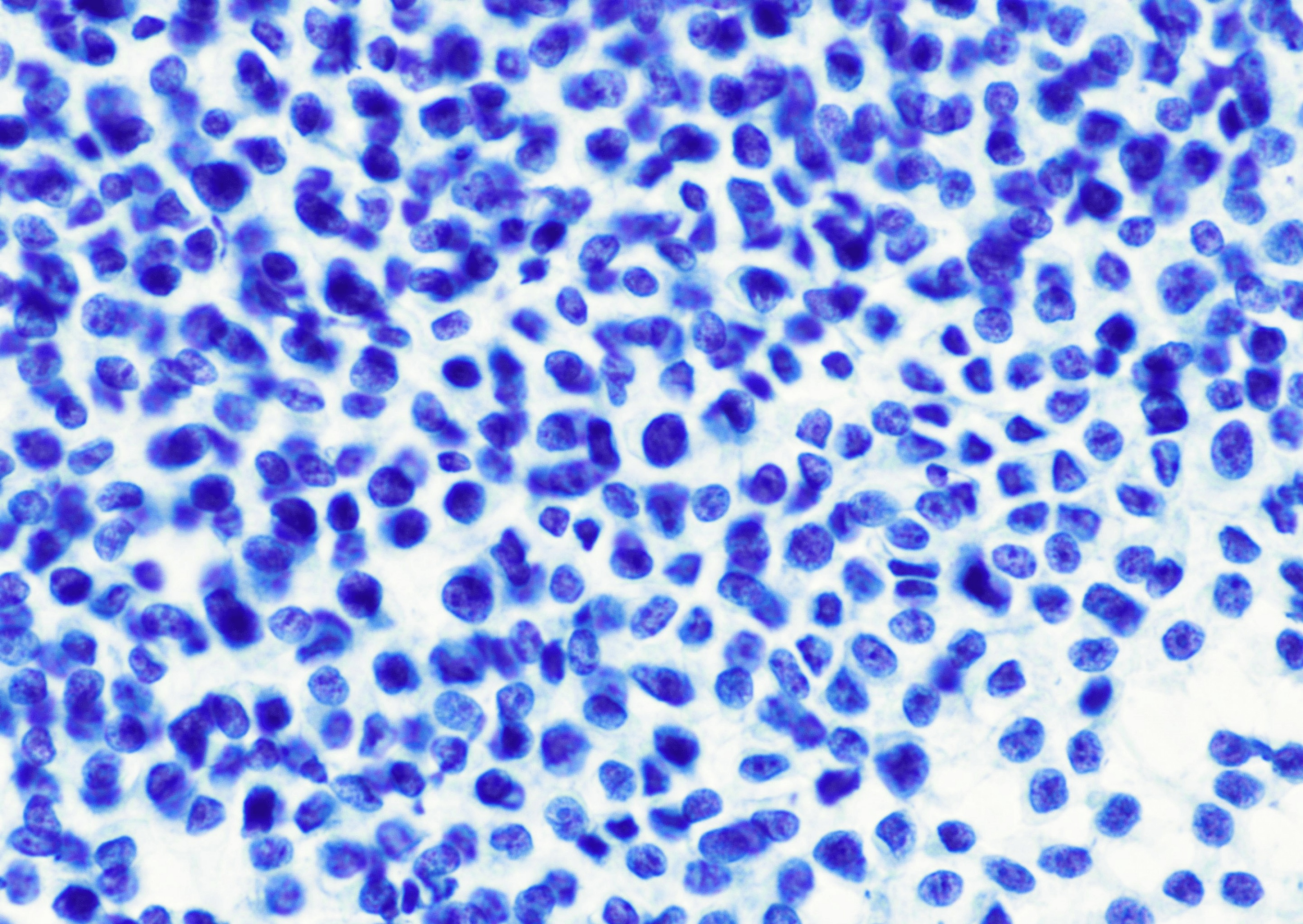
A Pap-stained touch preparation at 60× revealing dispersed small round cells with high nuclear-to-cytoplasmic ratios. Pap-stained touch preparation: 60×. Dispersed small round tumor cells with a high nuclear-to-cytoplasmic ratio and fine chromatin detail.

**Figure 5:**
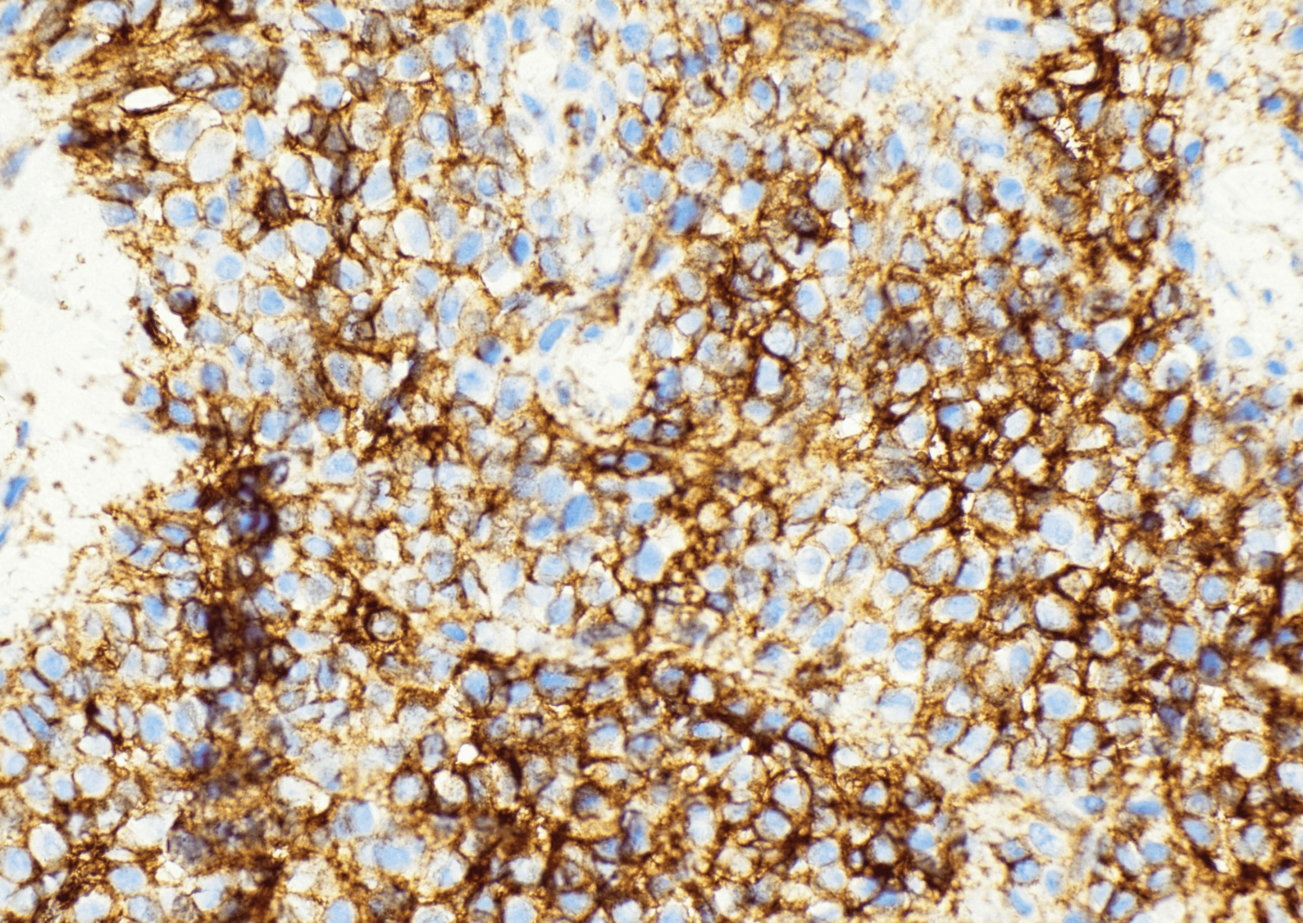
Diffuse strong membranous CD99 positivity at 40×, confirming the immunophenotypic profile characteristic of Ewing sarcoma. CD99 immunohistochemistry: 40×. Diffuse strong membranous positivity consistent with Ewing sarcoma. CD99: cluster of differentiation 99

Subsequent biopsy revealed a highly cellular neoplasm composed of uniform small round blue cells arranged in sheets and nests (Figures 2-4). At higher magnification, the tumor cells demonstrated round nuclei with finely dispersed chromatin and inconspicuous nucleoli, with cytoplasm ranging from scant to moderately abundant and pale, consistent with intracytoplasmic glycogen accumulation. Immunohistochemistry showed a diffuse membranous cluster of differentiation 99 (CD99) positivity (Figure 5) and negative staining for AE1/AE3, desmin, CD45, and S100. Targeted next-generation sequencing confirmed an *EWSR1*/*FLI1* fusion, establishing the diagnosis of ES.

The multidisciplinary oncology team initiated neoadjuvant chemotherapy with vincristine, doxorubicin, and cyclophosphamide alternating with ifosfamide and etoposide (VDC-IE) administered every 21 days for 12 weeks, followed by plans for local control and adjuvant therapy.

## Discussion

ES represents one of the prototypical small round blue cell tumors of bone and soft tissue, defined by *EWSR1* fusions with *ETS* family transcription factors, most commonly *FLI1* (~85%) and *ERG* (~10%) [1,2]. It is a highly aggressive malignant neoplasm that predominantly affects children and young adults, with a peak incidence in the second decade of life. Clinically, patients often present with localized pain and swelling, and systemic inflammatory manifestations such as elevated erythrocyte sedimentation rate or leukocytosis may be present, occasionally mimicking infectious or inflammatory conditions.

Although ES classically arises in the bone, a substantial proportion, approximately 15%-20%, occurs in extraosseous soft tissue, often adjacent to tendons or fascia [1]. These extraskeletal tumors may present without overt osseous involvement and are more likely to arise in unusual anatomic locations, contributing to diagnostic delay. The current case highlights this challenge, as the involvement of the ankle soft tissue represents a particularly rare presentation.

Histologically, ES is composed of uniform small round cells with scant cytoplasm, fine chromatin, and inconspicuous nucleoli, although variable cytoplasmic clearing related to intracytoplasmic glycogen accumulation may be observed [3]. Focal Homer-Wright rosette formation, reflecting limited neuroectodermal differentiation, may also be present. Historically, tumors demonstrating such features were classified as primitive neuroectodermal tumors (PNETs); however, advances in molecular pathology have established that ES and PNET represent a single biologic entity within the Ewing sarcoma family of tumors, unified by shared *EWSR1*-*ETS* gene fusions.

Immunohistochemistry is therefore essential in the diagnostic workup. Diffuse membranous CD99 and nuclear NKX2.2 (if performed) support the diagnosis of ES, while negative staining for AE1/AE3, desmin, and CD45 helps exclude carcinoma, rhabdomyosarcoma, and lymphoma, respectively [2]. However, these findings are not entirely specific, and definitive diagnosis relies on molecular confirmation. The presence of an *EWSR1*/*FLI1* fusion distinguishes ES from its major molecular mimics, including *CIC*-, *BCOR*-, and *NUTM1*-rearranged sarcomas [3].

The treatment of ES generally consists of multi-agent chemotherapy combined with surgical excision and/or radiation, with long-term survival rates approaching 70% for localized disease [4]. The identification of the *EWSR1*/*FLI1* fusion not only establishes the diagnosis but also may carry prognostic and therapeutic significance as targeted molecular approaches continue to evolve [5].

## Conclusions

This case highlights a rare presentation of extraskeletal ES arising in the soft tissues of the ankle, an unusual anatomic site that may delay clinical suspicion for this malignancy. While integrated morphologic, immunohistochemical, and molecular assessment remains essential for the accurate diagnosis of small round cell tumors, this report emphasizes the importance of maintaining awareness of ES at uncommon extraskeletal locations. The recognition of such atypical presentations is critical to avoid diagnostic delay and to facilitate timely, appropriate multimodality management.
